# Advance in sex differentiation in cucumber

**DOI:** 10.3389/fpls.2023.1186904

**Published:** 2023-05-17

**Authors:** Haiyan Luo, Huanchun Zhang, Huasen Wang

**Affiliations:** ^1^ Key Laboratory for Quality and Safety Control of Subtropical Fruits and Vegetables, Collaborative Innovation Center for Efficient and Green Production of Agriculture in Mountainous Areas of Zhejiang Province, Ministry of Agriculture and Rural Affairs, College of Horticulture Science, Zhejiang Agriculture and Forestry University, Hangzhou, China; ^2^ Engineering Laboratory of Genetic Improvement of Horticultural Crops of Shandong Province, College of Horticulture, Qingdao Agricultural University, Qingdao, China; ^3^ Hangzhou Lin’an District Agricultural and Rural Bureau, Hangzhou, China; ^4^ Yantai Institute of Agricultural Sciences, Yantai, China

**Keywords:** cucumber, sex differentiation genes, environmental conditions, phytohormones, regulatory mechanism

## Abstract

Cucumber belongs to the family Cucurbitaceae (melon genus) and is an annual herbaceous vegetable crop. Cucumber is an important cash crop that is grown all over the world. From morphology to cytology, from canonical genetics to molecular biology, researchers have performed much research on sex differentiation and its regulatory mechanism in cucumber, mainly in terms of cucumber sex determination genes, environmental conditions, and the effects of plant hormones, revealing its genetic basis to improve the number of female flowers in cucumber, thus greatly improving the yield of cucumber. This paper reviews the research progress of sex differentiation in cucumber in recent years, mainly focusing on sex-determining genes, environmental conditions, and the influence of phytohormones in cucumber, and provides a theoretical basis and technical support for the realization of high and stable yield cultivation and molecular breeding of cucumber crop traits.

## 1 Introduction

The sexes of plants are diverse and have evolved over a long period of biological evolution through a process of sexual differentiation. Plant sex is generally defined in terms of unisexual flowers, i.e., “if a flower or plant contains only stamens, it is male, and if it contains only pistils, it is female,” so studies on plant sex determination have focused on the regulation of unisexual flower development (Bai, 2015). In the evolution of plant sex types, hermaphroditic plants are considered to be the original sex types of land plants, while monoecious and dioecious unisexual flowers may have originated from hermaphroditic plants and emerged only after several evolutionary processes (Tanurdzic and Banks, 2004). Most angiosperms are hermaphrodites, producing only complete flowers. Sex determination is a developmental evolutionary process in which the formation of unisexual flowers facilitates heterosis and promotes genetic diversity (Chen et al., 2016). Over the past few decades, there has been a concerted effort to identify the determinants that control plant sex and to explore the evolutionary forces that drive plant sex variation (Diggle et al., 2011). As research continued, it was discovered that cucurbits cover most of the sex types of angiosperms, and some species have even evolved sex chromosomes (Ming et al., 2011).

Cucumber is a cucurbit family and cucumber genus of annual sprawling herb crops that have been commonly cultivated worldwide for a long time. Cucumber has a unique flavor, a crisp texture, is rich in nutrients, has low heat, and is enjoyed by people from all over the world. Cucumber is one of the seven major vegetables in China; its production area ranks first in the world, and it has important economic value (Zhang et al., 2018; Li H. et al., 2022). Cucumber yield is importantly related to sex differentiation. The cucumber sex system is complex and diverse, and the mechanism of male and female flower differentiation in cucumber is also intricate and complex. Several studies have attempted to characterize the molecular aspects of sex determination in cucumbers. At the early stage of cucumber flower development, the flower primordium is bisexual, including the initial form of the anther and pistil. In cucumber development, sex determination requires selective cessation of male or female progenitors (Bai et al., 2004; Pawełkowicz M. et al., 2019; Li Z. et al., 2022).

**Figure 1:**
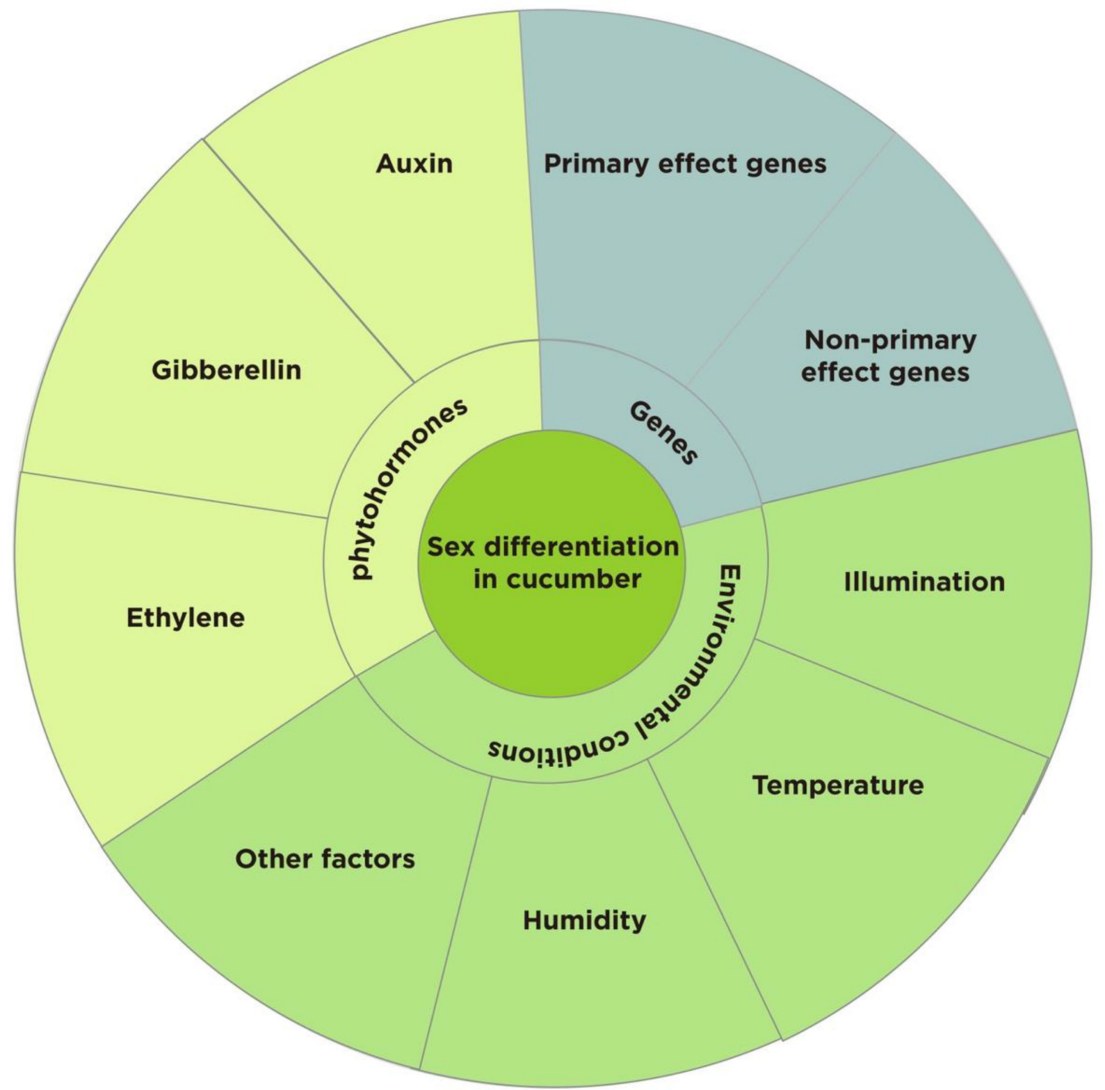
Factors influencing the sexual differentiation of cucumber.

As a model plant for studying sex determination in plants (Malepszy and Niemirowicz-Szczytt, 1991; Pawełkowicz M. E. et al., 2019; Li Z. et al., 2022), several studies have been conducted on the morphological anatomy and genetic basis of sexual differentiation in cucumber, but the genetic background of cucumber is narrow and the expression of sex types is complex and diverse (Grumet et al., 2022). This study provides a comprehensive analysis of the effects of sex-determining genes, environmental conditions, and phytohormones on sex differentiation in cucumber with the aim of providing a reference for future in-depth studies on sex differentiation, cultivation regulation, and molecular breeding of sex types in cucumber and related crops (**Figure 1**).

## 2 Types and processes of sexual differentiation in cucumber

### 2.1 Floral organ development model and sex differentiation in cucumber

Developmental genetics suggests that the developmental process of an individual is the orderly expression of a series of genes that are activated or turned off in a certain spatial and temporal sequence. Homozygous mutants in the model plants *Arabidopsis thaliana* and *Antirrhinum majus* for floral organ development provide ideal material for studying the molecular mechanisms regulating floral organ development. On this basis, scientists have proposed models for the development of various flower organs, the most typical of which is the “ABC model” of flower organ development (Bowman et al., 1991; Coen and Meyerowitz, 1991; Zhang et al., 2019). The ABC model represents three decisive genes affecting floral organ differentiation: class A genes regulate the formation of sepals; class B genes and class A genes jointly control the growth and development of plant petals; and class C genes can regulate carpel formation both individually and in cooperation with class B genes to control stamen production and development. In addition, class A and C genes are functionally antagonistic, which explains the phenomenon of homozygous heterotypic transformation of floral organs (Theißen, 2001; Krizek and Fletcher, 2005; Bowman et al., 2012; Ali et al., 2019). From the ABC model, we can see that the flower of the plant consists of four rounds of concentric circles, from the outside to the inside: sepals, petals, stamens, and carpels (Yanofsky, 1995; Alvarez-Buylla et al., 2010). Subsequently, a gene that defines the formation and development of ovules was found in *Petunia hybrida*, which is defined as the D gene (Angenent and Colombo, 1996). *SEEDSTICK (STK)*, *SHATTERPROOF1 (SHP1)*, and *SHP2* found in *Arabidopsis* have similar functions to those of D genes in petunias. In mutants of these three genes, the carpel of Arabidopsis substitutes for ovule formation (Pinyopich et al., 2003). In addition, class E genes were found and named in Arabidopsis, which can combine with other genes in the model to jointly maintain the development process of flower organs (Ditta et al., 2004). Sequencing and qualitative expression analysis of class A, class B, and class E MADS-box homologs in Australian shrubs and magnolia lilies added class D and class E genes to the ABC model, enriching and extending the model for controlling flower organ development to the “ABCDE” model (Kim et al., 2005; Shen and Wang, 2022).

As one of the most important classes of genes, MADS box genes regulate flower organ development in higher plants (Becker and Theißen, 2003). Class A, B, and C genes are homologous genes that can be transcribed into proteins, and the proteins encoded by these genes all contain a MADS cassette region. The STK and SEP genes belong to the MADS-box gene family. At present, several MADS box genes have been cloned from cucumbers, and the *ERAF17* gene is one of them (Filipecki et al., 1997; Ando et al., 2001; Hu and Liu, 2012; Zhou et al., 2019). *ERAF17* synthetic transcripts were induced in the growing points of cucumber plants of a monoecious variety and a purely female variety treated with ethylene glycol for 4 h. *ERAF17* expression was found to be localized in the flower buds of purely female plants and persistently expressed during female flower development, indicating that female flower formation was induced by ethylene, probably regulated by *ERAF17* expression in the apical flower buds of cucumber plants (Ando et al., 2001). *Cucumis melo*, a typical experimental material, has been found to be sexually differentiated by two loci, A and G. In melon, a gene encoding a C2H2-type zinc finger protein transcription factor, named *CmWIPI*, was found to control sex in melon together with another gene, *CmACS-7*, which controls ethylene synthesis in melon (Martin et al., 2009). In experiments with kiwifruit, a relative cytokinin regulatory gene, SyGI, was found on the Y chromosome, which suppressed pistil development in male kiwifruit flowers and is presumed to be a sex-determining gene in kiwifruit (Akagi et al., 2018).

### 2.2 Sex phenotype of cucumber

Cucumbers can produce female, male, and hermaphrodite flowers, and the three types of flowers are combined to produce a variety of sex systems. There are eight types: pure female plants, strong female plants, female whole plants, male and female whole plants, male and female plants, complete plants, male whole plants, and pure male plants (**Table 1**) (Wu et al., 2010). Cucumber plants have also been classified according to their floral phenotype into monoecious, strong female plants, all-female plants, all-male plants, intersexual flowering plants, male whole plants, female whole plants, trisexual flowering strains, and other sex system types (Che and Zhang, 2019). Early in cucumber bud development, both the androgynophores appear (Atsmon, 1960; Moraes, 2020); however, with the gradual growth and development of the plant, only one pistil primordium or stamen primordium can continue to develop, and the other gradually degenerates through apoptosis, thus forming a female or male flower (Perl-Treves, 2004). If the growth of both male and female flowers is not restricted, they will develop into bisexual flowers (Malepszy and Niemirowicz-Szczytt, 1991; Wang et al., 2022).

**Table 1:** Types of sex differentiation and features of cucumber.

Sex types	Features
Pure female plants	Female flowers at all nodes on the main stem and lateral branches.
Strong female plants	Most of the nodes on the main stem are female flowers and a very small proportion of nodes are male flowers.
Female whole plants	The main stem is preceded by a segment of complete flowers, followed by female flowers and complete flowers spaced apart.
Male and female whole plants	The main stem begins with a segment of male flowers, followed by a segment of male flowers, complete flowers and female flowers interspersed, and then female flowers predominate.
Monoecious	The main stem first has a segment of male flowering nodes, then the female flowers grow interspersed with male flowers, and some later have a segment of continuous female flowers with equal numbers of male and female flowers.
Complete plants	Staminate or bisexual flowers on main stem at first, followed by successive bisexual flowers, or possibly staminate flowers, or both staminate and bisexual flowers.
All-male plants	Male flowers at all nodes on the main stem and lateral branches.
Male whole plants	The main stem begins with a male flower, followed by a segment of complete (or female) flowers interspersed with male flowers, followed by complete flowers.

### 2.3 Sex differentiation process of cucumber

Through long-term research, scholars have found that the essence of sex differentiation in cucumbers is the differentiation of male and female flowers. The process of sexual differentiation in cucumbers is complex, as male and female flowers are initially hermaphroditic, and then selective abortion occurs when one of the male and female sex organs atrophies to form an unisexual male or female flower (Boualem et al., 2009). From a morphological point of view, the process of cucumber flower development can be divided into 12 stages and into two periods: the hermaphroditic stage and the differentiation stage (Bai et al., 2004; Wen et al., 2020). Stages 1–5 are when various organs in the flower, such as sepals, petals, stamen primordia, pistil primordia, and other floral meristematic tissues, begin to develop, and there is no obvious morphological difference between male and female flowers during this process, so it is called the hermaphroditic stage; in male flowers, the pistillate primordia are stagnant, anthers, and filaments start to develop at stage 6, anthers further expand at stage 7, locules differentiate at stage 8, microspore mother cells appear at stage 9, meiosis starts at stage 10, mononuclear pollen appears at stage 11, and finally mature pollen is formed at stage 12. In contrast, in female flowers, the carpel primordia begin to sprout and elongate at stage 6 and differentiate in the stigma and ovary at stage 7, followed by the elongation of the stigma, the development of ovules and beads at stage 8, the formation of macrospore cells at stage 9, meiosis at stage 10, the formation of embryo sacs at stage 11, and finally the maturation of all the appendage tissues at stage 12, with the stamen primordia lagging throughout the whole process (Hao et al., 2003; Zhang et al., 2021).

## 3 Genetic of sex differentiation in cucumber

In the long-term study of sex differentiation in plants, it was discovered that sex differentiation in cucumbers is jointly determined by multiple genes, the primary effect genes being *F/f*, *M/m*, *A/a*, *CsACO2/Csaco2*, and *G/g*. The non-primary effect genes: *In-F*, *Tr*, *m-2*, and *gy* play different degrees of modifying roles (**Table 2**). Five of these sex-linked master effector genes include three genes encoding 1-aminocyclopropane-1-carboxylic acid synthetase genes *F*, *M*, and *A*, an ACC oxidase gene that converts ACC to ethylene (*CsACO2*), and a transcription factor *G*. The main function of *CsACS2* is to inhibit stamen development; *CsACS11* stimulates carpel development; *CsWIP1* is inhibited by *CsACS11* as a carpel inhibitor; and *CsWIP1* can also inhibit the expression of *CsACS2* and *CsACO2* (Kubicki, 1969; Kubicki, 1974; Kubicki, 1980; Pawełkowicz M. et al., 2019). In genetic and regulatory research on sex differentiation, the master effect genes *F/f*, *M/m*, and *A/a* have been studied the most and are key genes in determining the direction of differentiation in cucumber. The *F* gene is partially dominant and controls female flower development and determines the female level of the plant; *Ff* is a strong female plant, *FF* is a full female plant; the *M* gene suppresses stamen development, while the *m* gene controls bisexual flower development invisibly; the *A* gene promotes carpel development, while the a gene controls invisibly. The sex type of cucumber is determined by the combination of three sex-determining genes: *F*_*M*_A/a is an all-female plant; *F*_*mm*A/a is a hermaphroditic plant; *ffM*_A_ is a dioecious plant; *ffmm*A_ is a male all-female plant; *ffM*_aa is a strong male plant; and *ffmmaa* is an all-male plant, where *F* has a dominant epistatic effect on *A* (Perl-Treves et al., 1998).

**Table 2:** Major genes controlling the sex of cucumber.

Classification	Gene	Function	Reference
Primary effect gene	*F/f*	Controls allogynous traits, interacts with a and m, and controls the development of female flowers.	Galun et al. (1962); Li et al. (2012); Pawełkowicz M. E. et al., 2019
	*M/m*	Inhibition of stamen development and maturation and control of the expression of hermaphroditic traits	Li et al. (2009); Boualem et al. (2009); Li et al. (2012); Pawełkowicz M. E. et al., 2019
	*A/a*	Control of allogamous trait expression, promotion of carpel development, and dominant male flower production	Boualem et al. (2015); Pawełkowicz M. E. et al., 2019
	*CsACO2/Csaco2*	Not specific for male and female flowers and promotes female flower carpels by synergistically producing ethylene	Kahana et al. (1999); Chen et al. (2016)
	*G/g*	The pure female gene, which represses carpel development by suppressing the expression of CsACS2 and CsACO2	Martin et al. (2009); Hu et al. (2017); Chen et al. (2016)
Non-primary effect genes	*In-F*	Enhancement of female expression in hermaphrodites, independent of the F gene, and female expression enhancer	Kubicki (1969)
	*Tr*	A co-dominant gene that eliminates the inhibitory effect of unisexual male pericarp development	Kubicki (1969)
	*m-2*	A male bisexual gene, independent of the m gene, regulates the development of the ovary into a normal bisexual flower	Kubicki (1974)
	*gy*	Controls all-female traits, is a highly female-expressed cryptic gene	Kubicki (1980); Pawełkowicz M. E. et al., 2019

### 3.1 *F/f* gene

In 1928, scientists uncovered the master gene driving the all-female phenotype of cucumber plants through genetic experimentation, marking the first discovery of the *F* gene. This gene promotes the early development of female flowers and enhances feminization by controlling the all-female characteristic in an incompletely dominant way (Rosa, 1928). In 1997, a gene with one additional copy than CsACS1 and a complete linkage to the *F* gene was identified in the all-female material and named *CsACS1G* (Trebitsh et al., 1997). Subsequently, in 2000, the spatial expression of *CsACS1/CsACS1G* was examined by Northern blot, and the results showed that the gene was expressed in the apical meristem of all-female plants but not in dioecious plants (Kamachi et al., 2000).

In 2015, a breakthrough was made in the study of *F/f* genes. Using 115 cucumber germplasm resequencing data, the *F* region of cucumber was analyzed comparatively and was found to be a 30.2 kb repeat unit that occurs in one, two, or four copies in all-female plants. In this analysis, two new genes, *A1* and *B1*, in addition to *CsACS1G*, were also identified for the first time in the *F* region (Zhang et al., 2015). In addition, in 2020, the phenomenon of “female loss” was found in no less than 0.11% of all-female cucumber plants, demonstrating the existence of some conserved and unstable CNP (copy number variant) variation structure of the *F* gene in cucumber (Li et al., 2020).

### 3.2 *M/m* gene

The *M* gene *(CsACS2)* encodes ACC synthase, a rate-limiting enzyme in ethylene biosynthesis. The earliest ACC synthase was not cloned for the first time until 1997, when the full-length sequence of CsACS2 was cloned and found to be expressed only in female flowers (Kamachi et al., 1997; Yuan et al., 2020); by 2001, it was found that the expression of the *CsACS2* gene was positively correlated with ethylene content, and the expression of *CsACS2* was higher in female plants than in monoecious and androgynous plants (Yamasaki et al., 2001); and in 2007, using *in situ* hybridization, it was found that *CsACS2* was expressed only in the carpels of female flowers, while male flowers were in the carpels of female flowers but not in male flowers. This finding was positively correlated with the regulation of the *M* gene (Saito et al., 2007).

In 2009, the *M* gene was cloned, and a SNP site (G97T) linked to the *M* gene was found in the first exon of the *CsACS2* gene, resulting in a heterozygous mutation of amino acid G33C (Li et al., 2009; Wang et al., 2022); in the same year, in other experiments, three types of heterozygous mutations of *CsACS2* were obtained in cucumber germplasm: G33C, P209S, and S399L, and the results of enzyme activity assay showed that the enzyme activity of *CsACS2* was reduced or even completely inactivated after the three types of mutations (Boualem et al., 2009). Finally, we concluded that *CsACS2* is an *M* gene that encodes an ACC synthase. In 2012, the positive expression mechanism between the *CsACS2* gene and ethylene was confirmed by ectopic expression of the *CsACS2* gene in tobacco, and the promoter of the *CsACS2* gene was activated by ethylene expression after transferring into tobacco (Li et al., 2012; Zhang et al., 2021).

### 3.3 *A/a* gene

Genetic research proved that *A/a* is entirely dominant and that a gene improves males. Genetic techniques revealed the existence of a recessive gene *A* regulating all-male features in plants as early as 1969 (Kubicki, 1969). Until 2015, a candidate gene for the *A* gene, *CsACS11*, also encoding ACC synthase, was cloned in cucumber using map cloning techniques. In all-male plants, a base deletion in exon 3 of *CsACS11* resulted in the premature termination of *CsACS11* translation. Two missense mutations, G39R and W58*, were identified by screening using the TILLING technique. After backcrossing with wild-type monoecious plants, *CsACS11* pure plants were mutated to be all-male, and female flowers were produced by applying ethylene to *CsACS11* mutants, thus further confirming that *CsACS11* is an *A* gene. *In situ* hybridization revealed that *CsACS11* was expressed in the 4-phase floral primordia of female flowers of monoecious plants and then in the inner and outer sides of the siliques of vascular bundles. *CsACS11* was first expressed in the 4-phase floral primordia of female flowers of monoecious plants, and then in the inner and outer sides of the vascular siliques and in the sieve tube of the carpels (Boualem et al., 2015).

### 3.4 CsACO2/Csaco2 gene

The *CsACO2* gene is capable of encoding an ACC oxidase. In 1999, *in situ* mRNA hybridization revealed that *CsACO2* has a specific expression pattern in different tissues and stages of flower development, mainly in the ovary and stamens (Kahana et al., 1999). By 2016, an all-male plant mutant recessively controlled by the *CsACO2* gene was identified using EMS mutagenesis, and this mutant trait. The population was constructed using a homozygous and all-male plant that encodes a rate-limiting enzyme in the ethylene synthesis pathway. *In vitro* activity assays showed that the mutant *CsACO2* enzyme activity decreased and ethylene release was correspondingly reduced while the all-male mutant was treated with ACC. The all-male mutant produced no female flowers after treatment with ACC. After ACC treatment, the male-only mutant did not generate any female flowers. In contrast, when ethylene was applied to the all-male mutant, no female flowers were formed. When mutant plants were treated with ethylene, continuous female flower nodes formed. Experiments revealed that in the absence of *CsACO2* enzyme activity, the plant was unable to catalyze the synthesis of sufficient ethylene by ACC and consequently was unable to stimulate the formation of female flowers. *In situ* hybridization showed that *CsACO2* expression started in the second floral primordium and overlapped with *CsACS11* expression at the carpels of the fourth floral primordium. The expression of *CsACO2* itself is not specific for male and female flowers and promotes female flower carpels by synergistically producing ethylene with *CsACS11* which is specifically expressed in female flowers (Chen et al., 2016).

### 3.5 *G/g* gene

The *G/g* gene was first identified in melon, which recessively controls the all-female phenotype of melon and encodes the C2H2 family of transcription factors. *CmWIP1* is the only transcription factor among the sex-determining genes studied so far (Martin et al., 2009). The *CsWIP1* mutant was created in 2017 using gene editing technology, and the mutant produces bisexual flowers in the lower node and continuous female flowers in the upper node (Hu et al., 2017). *CsWIP1* is a pure female gene that can be repressed by endogenous ethylene. *CsWIP1* can bind to the promoter of *CsACO2* to repress its expression and can also directly repress the expression of *CsACS2*. This suggests that *CsWIP1* plays a critical role in sex differentiation. This suggests that *CsWIP1* plays a critical role in sex differentiation (Chen et al., 2016).

## 4 Environmental factors impact the sex differentiation of cucumber

**Figure 2:**
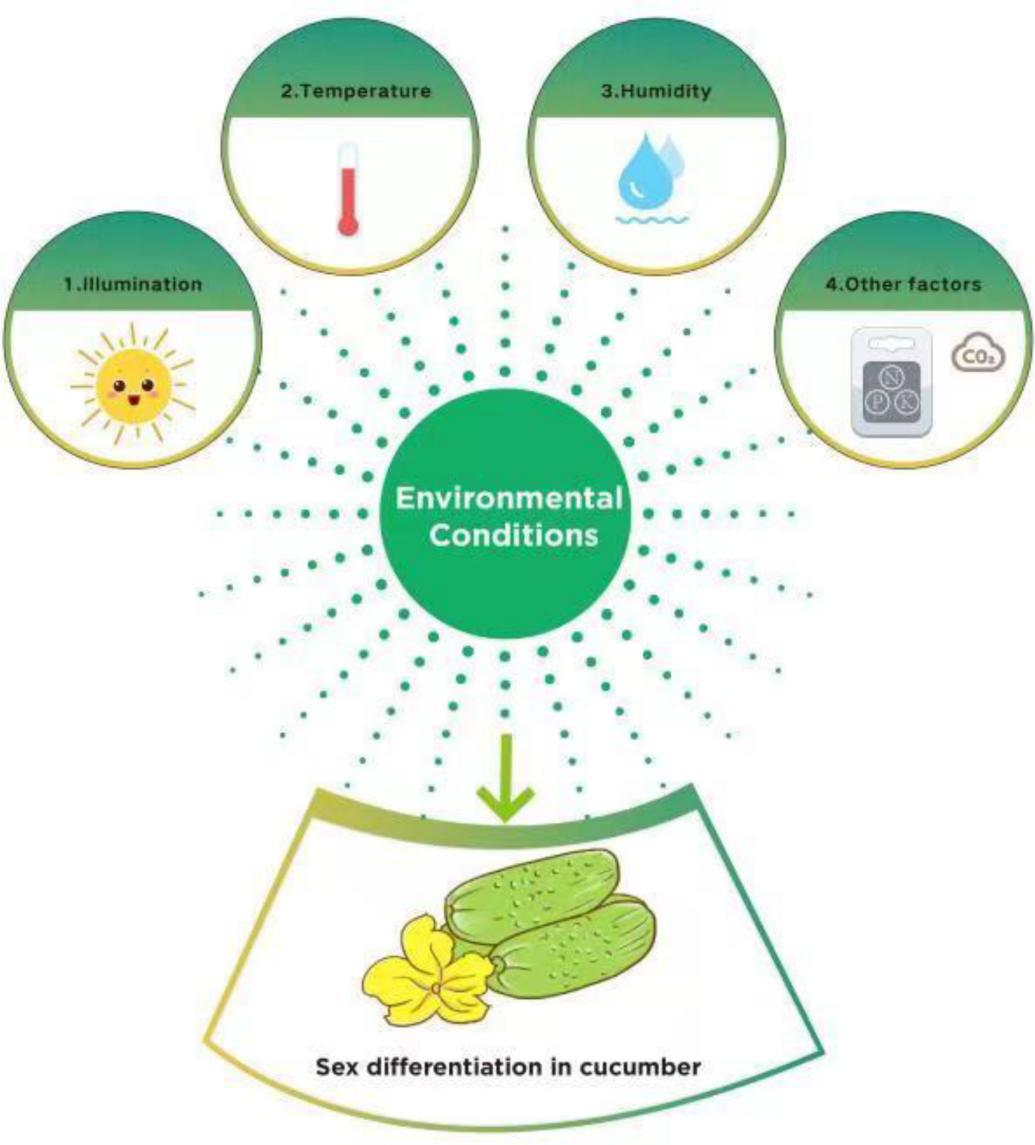
Environmental factors impact the sex differentiation of cucumber.

Environmental conditions such as illumination, temperature, humidity, and mineral nutrients are important factors impacting the sex differentiation of cucumbers. Environmental stress regulates the sexual differentiation of flowers and influences both the quality and yield of cucumbers (**Figure 2**).

### 4.1 Illumination

Light is a very important environmental factor that plays a role in the growth and development of plants. Photoperiod, light intensity, and light quality all play different roles in plant bud differentiation (Chory and Wu, 2001; Sidhu et al., 2021). Research shows that most cucumbers easily form female flowers in short photoperiods but more male flowers in long photoperiods (Cho et al., 2005; Tian et al., 2021). Short- and long-day-sensitive cucumbers have increased photosynthesis efficiency and an increased number of female flowers under short- and long-light conditions, respectively (Takahashi et al., 1983; Yang et al., 2022). The number of female flowers of long-day-sensitive cucumbers increased under high light intensity, while the shading of short-day-sensitive cucumbers was more conducive to the formation of female flowers (Cantliffe, 1981; Roy and Saran, 2019). Under the induction of red light, the long-day-sensitive cucumber produced more female flowers than other color light sources, such as white light and green light, and the number of female flowers increased with the extension of light interval time (Matsuo and Fukushima, 1970; Roy and Saran, 2019).

### 4.2 Temperature

Temperature had a great influence on the sex differentiation of cucumber, mainly because it significantly affects the node position of female flowers and the ratio of male to female flowers. Low temperatures can promote female flower differentiation, while high temperatures can induce male flower formation. Under certain high temperatures, it can promote the development of cucumber flower organs and flowering in advance, but if it encounters continuous high temperatures for a long time, it will lead to male flower buds falling and not flowering (Chen L. et al., 2021). Under the action of high temperature, the percentage of female flower nodes in cucumber decreased, and the smaller the seedling age was, the greater the impact of high temperature. At 35°C, the pollen tube length of cucumber was significantly reduced, and at 40°C, the pollen germination rate was significantly lower than that at 28°C (Miao et al., 2000; Cheng et al., 2023).

### 4.3 Humidity

Humidity is closely related to the sex differentiation of cucumbers. Proper humidity will reduce the node position of the first female flower and increase the number of female flowers (Ito and Saito, 1960; Maeda and Ahn, 2021). Research shows that higher soil and air humidity are conducive to the female formation of plants. In a certain range, the higher the soil water content is, the more conducive it is to the formation of cucumber female flowers; the higher the air humidity is, the better the differentiation of cucumber female flowers (An et al., 2020). The development of female organs is easier than that of males in the presence of high air humidity. At the stage of bud differentiation, the increase in the number of flowers, especially the number of female flowers, is more facilitated by suitable air humidity.

### 4.4 Other factors

Different classes and levels of mineral nutrients also affect the outcome of sex differentiation in cucumber (Sarkar et al., 2019). Nitrogen nutrition is beneficial to promote the formation of female flowers. Under the condition of strong photosynthesis and good carbon nutrition, the supply of nitrogen can increase the number of female flowers on the main stem, but too much nitrogen is detrimental to the differentiation of female flowers; phosphorus fertilizer can promote the reproductive growth of cucumber plants and promote the differentiation of female flowers; potassium nutrition induces the formation of male flowers and is detrimental to the differentiation of female flowers. Nitrogen and phosphorus are applied separately to favor the formation of female flowers, but when potassium is applied separately, it favors the formation of male flowers instead (Vaudo et al., 2022). Increasing the amount of carbon dioxide in the air increases the photosynthetic efficiency of cucumber plants and promotes the differentiation of female flowers (Askary et al., 2020). After several experiments, the effect of short-time low temperature treatment (23°C during the day/15°C at night) on cucumber females was determined by whole-genome bisulfite sequencing (WGBS) of the stem tip. The effect of temperature on transposon-related RNA-directed DNA methylation was found by whole genome methylation sequencing, mRNA sequencing, and sRNA sequencing analysis of stem tip parts. The effect of temperature on transposon-associated RNA-directed DNA methylation mechanisms was found to be significant, resulting in substantial CHH-type cytosine demethylation (Lai et al., 2017). The results showed that blue light induced stronger induction of female flowers than other light substances, and the blue light-induced sex expression in female flowers was closely related to the blue light-induced changes in abscisic acid, growth hormone, gibberellin (GA), photosynthesis, starch, and sucrose metabolic pathways (Zhou et al., 2018).

## 5 Hormonal regulation of sex differentiation in cucumber

The most unique part of the study of sex determination in cucumbers is that the ratio of male to female flowers in the plant is influenced by phytohormones. These properties not only provide an efficient production method for cucumber cultivation but also provide a reliable way to understand the regulatory mechanisms of unisexual flower development. Among the plant growth hormones, ethylene, GA, and IAA have the greatest influence on the sex expression of cucumber. Among the plant growth hormones, ethylene, GA, and IAA have the greatest effect on sex expression in cucumbers. Among them, ethylene and IAA induce the growth and development of female flowers in cucumber, while GA promotes the development of male flowers and hinders the development of female flowers in cucumbers.

### 5.1 Ethylene

Ethylene is a phytohormone commonly used in cucumbers to promote the formation of female flowers (Zhang et al., 2017). It determines the sexual development of individual floral meristems of cucumber and promotes the formation of female flowers (Martínez and Jamilena, 2021; Cebrián et al., 2022). In a study by Garcia et al., two new ethylene-insensitive mutations (*etr1a-1* and *etr1b*) were identified that blocked the female flowering transition in cucumber; this allowed the plant to produce male flowers (staminate flowers) indefinitely (García et al., 2020). Other ethylene biosynthesis genes of ACC oxidase, such as *CsACO2* and *CsACO3*, are also involved in cucumber sexual expression (Chen et al., 2016); however, the transcript levels of *CsACO2* and *CsACO3* were negatively correlated with female flowering (Kahana et al., 1999). In addition, the ethylene receptor CsETR1 plays an important role in the inhibition of female cucumber flower stamens through the induction of DNA damage (Wang et al., 2010). The cucumber nuclease-encoding gene CsCaN induces DNA damage in the anther primordia in response to ethylene signaling, resulting in stalled development of the anther primordia and the formation of female flowers (Gu et al., 2011). Inhibition of acetylenic biosynthesis and acetylenic signaling results in a decrease in the number of female or bisexual flowers (Atsmon and Tabbak, 1979; Takahashi and Jaffe, 1984) and an increase in the number of male flowers (Ando et al., 2001). In the female flower experiment, ethylene signaling activated CsERF31 through CsEIN3, and then CsERF31 stimulated CsACS2, thus promoting the development of female flowers (Pan et al., 2021).

The “single hormone hypothesis” was widely accepted to explain the ethylene-mediated differentiation of unisexual flowers in cucumber, suggesting that ethylene in cucumber suppresses male flowers and induces female flowers by regulating the expression levels of *F* and *M* (Trebitsh et al., 1997). However, with the cloning of the *M* gene, this hypothesis was soon revised, and a positive feedback mechanism was proposed for the *M*-mediated regulation of ethylene (Li et al., 2008; Li et al., 2012). In 2018, it was discovered that *CsERF31* directly binds to the promoter of *M* and promotes its expression, and on this basis, an “ethylene-*CsERF31*-*M*-ethylene” positive feedback mechanism was proposed: during female cucumber pistillate differentiation, *F* produces ethylene to promote pistil development, while *CsERF31* responds to the ethylene signal and activates *M*, and *M* starts its positive feedback activation expression through *CsERF31*. Ethylene downregulates *CsAP3* and *CsPI* by stimulating *CsSUP*, resulting in inhibition of *CsETR1* expression and an inability to downregulate *CsERF31*. In this process, the development of stamen primordia is hindered and the production of female flowers is promoted. This model explains the development and evolution of cucumber unisexual flowers from the perspective of the developmental fate of pistil and stamen primordia, but it is not suitable for monoecious cucumbers because there is currently no evidence that *M* is activated by *F* in the predetermined female buds (Pan et al., 2018).

### 5.2 Gibberellin

Gibberellin (GA) is an important factor in cucumber sex differentiation that can promote male flower differentiation and hinder female flower differentiation (Staub and Bacher, 2023). In 1960, gibberellin was first discovered to induce male flowers in female line plants, thus solving the problem of female line selection and reproduction (Li et al., 2019). GA yield was higher in male hermaphroditic cucumber plants than in pure female and monoecious plants (Hemphill Jr et al., 1972; Kim et al., 2021). In subsequent experiments, it was determined that GA3 spraying increased the ratio of males to females in monoecious cucumber plants and induced the formation of male flowers in female plants (Pike and Peterson, 1969; Yadav et al., 2022). In *Arabidopsis* and rice, the GA signaling pathway is involved in the development of stamens and anthers in hermaphroditic plants (Aya et al., 2009; Song et al., 2013; Chen M. et al., 2021). GA3, GA4, GA7, and GA13 are all functional in the induction of male flowers in female cucumber plants (Wittwer and Bukovac, 1962; Clark and Kenney, 1969; Zhu et al., 2021); GA4 and GA7 were sprayed at 50 mg kg^−1^, while GA3 was sprayed at 100 mg kg^−1^, and the former produced more male flowers (Pike and Peterson, 1969).

### 5.3 Auxin

Auxin (IAA) is a key hormone that determines growth and developmental dominance and seriously affects cucumber sex differentiation (Qin et al., 2021). As early as the 1950s and 1960s, it was found that a certain concentration of IAA could increase the female/male flower ratio and promote female flower differentiation in cucumber under *in vitro* conditions, while the growth hormone content in all-female cucumber plants was higher than that in dioecious homozygous plants (Galun et al., 1965; Rudich et al., 1972; Shekari et al., 2019). In female cucumber flowers, the IAA content was higher than that in male flowers, and the IAA content determined the direction of sex differentiation in cucumber (Chen et al., 2002; Qin et al., 2021). It was shown that the male buds of cucumber could be converted into female flower buds by adjusting the IAA concentration (Galun et al., 1962; Cebrián et al., 2022). *CsSPL* in cucumber regulates anther and ovule development through interaction with the growth hormone-corresponding factor *CsARF3* (Liu et al., 2018).

## 6 Perspectives

Sex differentiation in cucumbers is regulated by genes, environmental factors, and hormones, which is a very complex and long evolutionary process. The study of sex differentiation is important to guide the selection of stable female lines for breeding and production in cucumber. With the continuous improvement of genomics, molecular genetics, and gene mining technologies, the mechanism of sex differentiation in cucumber has made great progress, and the framework of the sex differentiation regulatory network has been gradually clarified, but the diversity and variability of cucumber sexes seem to be difficult to comprehensively describe by the changing pattern of a single metabolic pathway. In the future, by studying the transcriptional and expressional regulatory mechanisms of sex determination genes, we will clarify the specific relationship between sex determination and classical hermaphroditic flowers and thus improve the gene regulatory network of sex determination in cucurbits. At the same time, by integrating the latest multidisciplinary research results and technologies in genomics and establishing the internal and external multifactorial and multidirectional interaction regulatory network, it is expected that a more in-depth and comprehensive analysis of the molecular genetic mechanism of sex will provide an important theoretical basis and technical support for high-yielding and stable cultivation and phenotypic genetic breeding of cucumber.

## Author contributions

HW, HL, and ZH conceived and designed the review. HL wrote the manuscript, and HW proposed revisions to the manuscript. All authors contributed to the article and approved the submitted version.
